# Evaluation of Renal Stiffness Using Shear Wave Elastography in Patients with Inactive Lupus Nephritis

**DOI:** 10.3390/jcm15031273

**Published:** 2026-02-05

**Authors:** Esin Olcucuoglu, Halil Tekdemir, Gulsah Soyturk, Mihriban Alkan, Alperen Sefa Toker, Hatice Ecem Konak, Mercan Tastemur, Kevser Orhan

**Affiliations:** 1 Department of Radiology, Ankara Bilkent City Hospital, Ankara 06800, Turkey; halil.tekdemir@saglik.gov.tr (H.T.); mihriban.alkan@saglik.gov.tr (M.A.); alperensefa.toker@saglik.gov.tr (A.S.T.); 2 Department of Rheumatology, Ankara Bilkent City Hospital, Ankara 06800, Turkey; gulsah.soyturk@saglik.gov.tr (G.S.); haticeecem.konak@saglik.gov.tr (H.E.K.); kevser.gok1@saglik.gov.tr (K.O.); 3 Department of Geriatrics, Ankara Bilkent City Hospital, Ankara 06800, Turkey; mercan.tastemur@saglik.gov.tr; 4 Department of Physical Medicine and Rehabilitation, Ankara Yıldırım Beyazıt University School of Medicine, Ankara 06800, Turkey

**Keywords:** systemic lupus erythematosus, lupus nephritis, Doppler ultrasonography, shear wave elastography, fibrosis

## Abstract

**Background/Objectives:** Lupus Nephritis (LN) is a major complication of Systemic Lupus Erythematosus (SLE) leading to significant morbidity. While biopsy is the gold standard, non-invasive tools are needed for longitudinal monitoring. This study aims to evaluate the diagnostic utility of Shear Wave Elastography (SWE) in detecting subclinical renal damage (fibrosis) in SLE patients with a history of LN who are currently in clinical remission (inactive disease), and to compare its efficacy with Doppler ultrasonography (DUS). **Methods:** This cross-sectional study included 80 SLE patients and 41 age- and sex-matched healthy controls. Crucially, all SLE patients were in the clinically inactive disease (SLEDAI-2K < 6) at the time of evaluation. Patients were stratified into two groups: those with a history of LN (LN Group, n = 37) and those without (Non-LN SLE Group, n = 43). Strict exclusion criteria were applied to eliminate non-SLE renal comorbidities. Renal parenchymal stiffness (kPa) was measured using SWE, and the renal resistive index (RI) was assessed using DUS. SWE findings were correlated with renal function tests and disease activity scores. **Results:** Despite being in clinical remission, the LN group exhibited significantly higher renal stiffness values (Median: 1.60 kPa) compared to the non-LN SLE group (1.40 kPa, *p* < 0.001) and healthy controls (1.32 kPa, *p* < 0.001). No significant difference was observed between the non-LN SLE group and controls. Unlike SWE, renal RI values showed no statistically significant difference among the groups (*p* > 0.05). Correlation analysis revealed that renal stiffness was positively associated with prior serum creatinine and disease activity (SLEDAI-2K), and negatively associated with eGFR. **Conclusions:** SWE is superior to DUS (RI) in detecting renal parenchymal changes in LN patients. The persistence of elevated stiffness during the inactive disease suggests that SWE captures cumulative chronic damage (remodeling and fibrosis) rather than just acute inflammation. Consequently, SWE holds promise as a non-invasive surrogate for monitoring disease chronicity in SLE patients.

## 1. Introduction

Systemic lupus erythematosus (SLE) is a chronic, inflammatory autoimmune disease characterized by the immune system attacking the body’s own tissues. It can affect multiple organ systems, including the skin, musculoskeletal system, kidneys, hematologic system, central nervous system, and lungs. SLE presents with a wide variety of clinical and laboratory findings and develops as a result of the interaction of genetic, environmental (e.g., drugs, UV exposure, viral infections), and hormonal factors. It is well known that SLE is seen more in women and epidemiologically, the disease is nine times more common in women than in men [1]. Consequently, research focusing on SLE carries significant implications for advancing women’s health.

Renal involvement in SLE, known as lupus nephritis (LN), is detected in approximately 25–38% of patients, often presenting as the initial symptom [2,3,4]. LN is the most common secondary glomerular disease and represents one of the most serious organ manifestations of SLE [2]. The pathological mechanism of LN involves the deposition of immune complexes, which trigger a cascade of inflammatory reactions, ultimately leading to kidney damage and fibrosis [5]. This inflammatory cascade leads to widespread nephron loss during acute diseases, which may progress to irreversible renal dysfunction if therapeutic intervention is not initiated promptly [6]. Approximately 10% of patients with LN develop end-stage renal disease (ESRD) within 10 years [1,7]. Epidemiological studies, including recent long-term cohorts, indicate that LN is a significant predictor of poor outcome, increasing the mortality risk by more than six-fold compared to the general population [1,2,3,4,7,8].

Early diagnosis and continuous monitoring of LN are crucial for improving the disease course and preventing potential kidney damage. Diagnosis primarily relies on patient history, physical examination, urinalysis (proteinuria, hematuria, casts), serum creatinine/urea levels, and serological markers such as antinuclear antibody (ANA) and anti-double-stranded DNA (anti-dsDNA) antibodies, a specific marker for LN activity. While ultrasonography (US) is widely used to assess kidney structure and Doppler ultrasonography (DUS) is preferred to examine renal blood flow, these methods are often insufficient for a definitive diagnosis [9,10].

Renal biopsy remains the gold standard for confirming LN diagnosis, determining the pathological type and severity, guiding treatment selection, and evaluating treatment response. Although generally safe, biopsy is an invasive procedure that can lead to complications such as bleeding, infection, arteriovenous fistulas, pain, and rarely, organ damage [11].

Although renal biopsy remains the gold standard for evaluating renal tissue, its invasive nature limits its clinical application in certain settings and patient populations. Consistent with the shift toward patient-centered care, non-invasive and radiation-free diagnostic modalities are increasingly prioritized. Shear Wave Elastography (SWE) has emerged as a valuable, accessible, and quantitative alternative for assessing tissue stiffness, with growing research focusing on its utility in glomerular disorders and renal function [12]. Distinct from traditional strain elastography, which relies on operator-dependent manual compression, SWE utilizes focused ultrasonic Acoustic Radiation Force Impulses to generate transverse shear waves within the tissue [13]. The propagation speed (Vs) of these waves is tracked in real-time; since shear waves travel faster in stiffer media, the velocity is directly proportional to tissue stiffness. This stiffness is quantified as Young’s modulus (E) and expressed in kilopascals (kPa). Crucially, SWE minimizes operator-induced variability, offering more standardized measurements than conventional methods [13]. In clinical practice, SWE is already established for assessing liver fibrosis, differentiating benign from malignant breast lesions, and objectively measuring structural changes in musculoskeletal tissues [14,15]. Notably, studies correlating SWE with renal biopsy have demonstrated that elasticity values track closely with the degree of fibrosis, showing progressive increases in stiffness concomitant with advancing chronic kidney disease (CKD) stages [16,17].

This study aims to assess the diagnostic performance of SWE as a non-invasive adjunct to renal biopsy in adult SLE patients. We specifically focused on comparing renal SWE values between patients with a history of LN and those without LN, all of whom were in clinical remission. This approach allows us to evaluate SWE’s utility in detecting potential subclinical renal alterations in patients with a history of LN, even during clinical remission. To our knowledge, this represents the first investigation within the existing literature to evaluate renal elasticity specifically in adult SLE patients during clinical remission.

## 2. Materials and Methods

### 2.1. Setting

The study was conducted at a large-scale medical campus consisting of six hospitals with a total capacity of 4000 beds and providing outpatient services to more than 30,000 patients daily. The research was carried out in the rheumatology and radiology departments, both of which operate as specialized tertiary healthcare centers. 

### 2.2. Ethical Approval

The study was approved by the Medical Ethics Committee of the Hospital (Approval Number: 2023-K093-01).

### 2.3. Study Design and Participants

This cross-sectional study was conducted in accordance with the Declaration of Helsinki. The medical records of patients diagnosed with SLE at our clinic between June 2024 and January 2025 were screened. The diagnosis of SLE was based on the European League Against Rheumatism and the American College of Rheumatology criteria [18]. Adult patients presenting with clinically inactive disease who met the inclusion criteria were referred from the rheumatology outpatient clinic for SWE evaluation.

The study included 121 participants: 80 patients with SLE and 41 age- and sex-matched healthy volunteers. Based on clinical evaluation, laboratory findings, and/or renal biopsy results, the SLE patients were categorized into two subgroups:

- Group 1 (LN Group): The diagnosis of LN was confirmed by renal biopsy in 17 (45.9%) patients (Class III–IV: n = 13; Class II–V: n = 4). In the remaining 20 patients, the diagnosis was established based on clinical and laboratory findings (proteinuria > 0.5 g/day or active urinary sediment) consistent with American College of Rheumatology and Systemic Lupus International Collaborating Clinics criteria [18,19]. This clinical diagnosis group includes 5 patients whose biopsy specimens were reported as ‘insufficient sample’ and 15 patients who did not undergo biopsy due to refusal or unavailability of reports.
- Group 2 (Non-LN SLE Group): SLE patients with no history or current clinical/laboratory evidence of renal involvement (n = 43).
- Group 3 (Control Group): Healthy volunteers with no history of systemic or renal disease (n = 41).

Clinical Assessment and Activity Definitions Systemic and renal disease activity were assessed using the Systemic Lupus Erythematosus Disease Activity Index 2000 (SLEDAI-2K) [20]. Clinical activity was defined as a total SLEDAI-2K score ≥ 6. In this retrospective analysis, patients in the non-LN SLE group exhibiting the ‘Clinically Active Serologically Inactive’ pattern—characterized by clinical-serological discordance—were classified as active. Active LN was defined by the presence of renal descriptors (proteinuria > 0.5 g/24 h, hematuria, pyuria, or urinary casts), each contributing 4 points to the index [20].

Crucially, all patients were referred for SWE during their inactive disease, and all imaging procedures were performed on the same day. The inactive disease was defined as a total SLEDAI-2K score < 6 with a complete absence of renal activity. This required stable or improved serum creatinine, a ‘quiet’ urinary sediment (<5 RBC/hpf and <5 WBC/hpf), and clinically insignificant proteinuria (<0.5 g/24 h). In this study, ‘inactive’ LN was defined primarily by clinical renal quiescence. Specifically, included patients had no clinical manifestations of active nephritis at the time of evaluation. Although the majority of the cohort was also serologically quiescent, patients demonstrating isolated serological activity (e.g., elevated anti-dsDNA or hypocomplementemia) in the absence of clinical renal findings were not excluded. This approach allows for the inclusion of patients fitting the ‘Serologically Active Clinically Quiescent (SACQ)’ phenotype described by Gladman et al. [21]. All patients in this study had been on a stable maintenance dose of immunosuppressants or were off therapy for at least three months prior to SWE assessment.

Patients were recruited from a single collaborating outpatient clinic based on strict inclusion and exclusion criteria. The study included individuals aged >18 years who met the 2019 European League Against Rheumatism/American College of Rheumatology classification criteria for SLE and provided voluntary consent on the day of examination. To ensure sample homogeneity, only patients with inactive SLE (with or without inactive LN) were enrolled.

**Figure 1:**
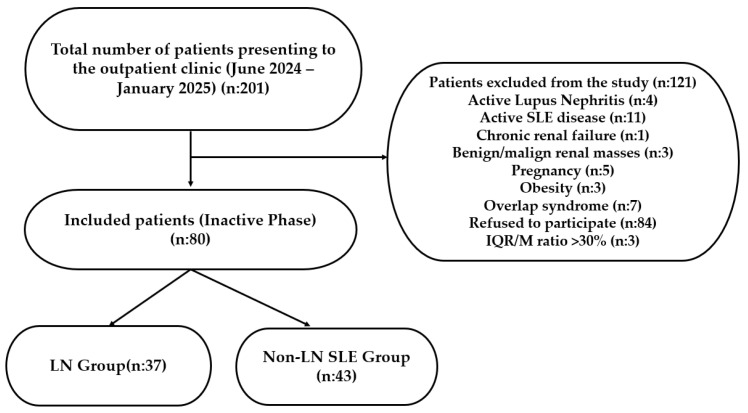
Flowchart detailing inclusion and exclusion of patients.

To eliminate potential confounding factors affecting renal stiffness, strict exclusion criteria were applied: active SLE or LN flare, chronic renal failure, benign or malignant renal masses, structural renal anomalies, pregnancy, obesity, and overlap syndromes. Although patients with comorbidities such as diabetes and hypertension were eligible, they were included only if they were euglycemic and normotensive under treatment; uncontrolled cases were excluded to ensure a comparable baseline. Furthermore, elastography measurements meeting objective criteria for poor image quality (IQR/M ratio > 30) were discarded. The non-LN SLE group was age- and sex-matched to the LN group. Of the 201 eligible patients invited, 80 consented to participate. The detailed patient selection process is illustrated in Figure 1.

### 2.4. Clinical and Laboratory Assessment

Demographic data, clinical characteristics, and disease duration were recorded for all participants. Detailed laboratory evaluations included complete blood count, erythrocyte sedimentation rate, C-reactive protein (CRP), fasting blood glucose, and liver function tests (Alkaline Phosphatase, Alanine Aminotransferase, Aspartate Aminotransferase). Renal function was assessed via serum creatinine, blood urea nitrogen levels, and estimated glomerular filtration rate (eGFR). Immunological markers, including antinuclear antibodies (ANA, anti-dsDNA), complement component 3 and 4 levels, were quantified using indirect immunofluorescence and radial immunodiffusion techniques, respectively. Disease activity was calculated using the Systemic Lupus Erythematosus Disease Activity Index 2000 (SLEDAI-2K) score. To evaluate the relationship between tissue stiffness and inflammatory history, SWE values obtained during the current inactive disease state were compared with the SLEDAI-2K scores and laboratory findings recorded retrospectively from the patients’ most recent active disease flare.

### 2.5. Sonographic Evaluation: Doppler Ultrasonography and Shear Wave Elastography

All sonographic examinations were performed by a single radiologist with more than 10 years of experience who was blinded to the participants’ clinical data and group assignments. A Logiq S8 ultrasound system (GE Healthcare, Chicago, IL, USA) equipped with a 3.5 MHz convex transducer (C1-5-D) was used for all evaluations. Measurements were obtained after at least 6 h of fasting and with an empty bladder to standardize intra-abdominal pressure.

**Figure 2:**
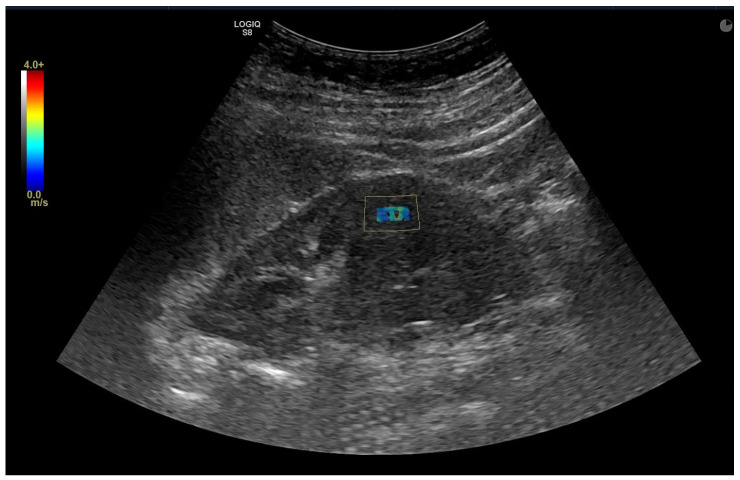
A Shear Wave Elastography (SWE) image was obtained from a patient in the control group, specifically from the middle part of the cortex of the kidney’s long axis, perpendicular to the skin, and at the closest point.

Renal parenchymal stiffness was measured quantitatively in kilopascals (kPa). While the patient was lying on their side and holding their breath, measurements were taken from the middle part of the cortex of the kidney’s long axis, perpendicular to the skin and at the closest point (Figure 2). No pressure was applied to the probe during measurements. The measurement depth was standardized between 3 and 5 cm to avoid depth-related artifacts. The Region of Interest (ROI) with a diameter of 10 mm was placed perpendicular to the renal capsule in the middle part of the renal cortex, avoiding medullary pyramids and vascular structures. To ensure data quality, only measurements with a stable shear wave color map and an interquartile range to median ratio (IQR/M) < 30% were included. To ensure intra-observer reliability, the mean of three consecutive measurements was used, and the coefficient of variation was calculated to assess measurement stability.

The renal Resistive Index (RI), an indicator of renal vascular resistance, was measured by DUS from the interlobar arteries at the middle part of the renal cortex of both kidneys. Participants were rested in a supine position for at least 10 min before the examination to stabilize the heart rate and renal blood flow. The average of three measurements was recorded for each kidney.

Poor image quality was objectively defined based on device-specific Quality Map assessments and measurement variability metrics. Specifically, measurements were excluded if the Quality Map demonstrated inconsistent color filling, indicating poor shear wave propagation, or if the interquartile range (IQR) of the 10 valid acquisitions exceeded 30% of the median value (IQR/M > 30%). Furthermore, considering the technical limitations of the GE Logiq E8 convex probe regarding depth penetration, patients with a skin-to-kidney distance exceeding 6 cm were excluded. This depth threshold was applied to ensure an adequate signal-to-noise ratio and to prevent depth-related artifacts commonly associated with obesity.

### 2.6. Statistical Analysis

The statistical analyses of the study were performed using the IBM SPSS Statistics (Version 22.0, IBM Corp., Armonk, NY, USA) statistical software. The distribution of continuous variables was assessed using the Shapiro–Wilk test; variables that did not meet the assumption of normal distribution were summarized using the Interquartile Range (IQR), and nonparametric tests were preferred for group comparisons. The Kruskal–Wallis test was applied for three-way comparisons based on the levels of kidney involvement. The Mann–Whitney U test was used to compare groups with and without lupus nephritis. For categorical variables, group differences were assessed using the Pearson chi-square test; when the expected cell frequency was <5, Fisher’s exact test was preferred. Relationships between continuous variables were examined using Pearson correlation analysis; Spearman rank correlation was considered as an alternative in the presence of assumption violations or obvious outliers. A *p*-value < 0.05 was considered statistically significant.

## 3. Results

### 3.1. Demographic and Clinical Characteristics

The study included a total of 121 participants: 80 patients with SLE and 41 healthy controls. The majority of participants in both groups were female: 76 (95%) in the SLE group and 39 (95.1%) in the healthy control group. The median age was 44.1 years for the SLE group and 42.2 years for the control group. There were no statistically significant differences between the groups in terms of age or gender distribution (*p* > 0.05).

The SLE patients were divided into two subgroups: 37 patients (46.2%) with LN (LN Group) and 43 patients (53.8%) without renal involvement (non-LN SLE Group). Demographic data and comorbidity profiles, including hypertension, diabetes, and coronary artery disease, were similar across the LN, non-LN SLE, and control groups (*p* > 0.05) (Table 1).

### 3.2. Comparison of Sonographic Findings

Renal parenchymal stiffness values measured by SWE showed significant differences among the three groups for both the right (H(2) = 30.10, *p* < 0.001) and left kidneys (H(2) = 31.67, *p* < 0.001). The LN group exhibited significantly higher renal stiffness values compared to the non-LN SLE group (Right: 1.6 vs. 1.4 kPa, *p* < 0.001; Left: 1.7 vs. 1.40 kPa, *p* = 0.001). No statistically significant difference was observed in renal stiffness between the non-LN SLE group and healthy controls (*p* > 0.05 for both kidneys).

In contrast to SWE findings, RI values did not differ significantly among the three groups (Right: *p* = 0.083; Left: *p* = 0.328). Pairwise comparisons also confirmed no significant difference in RI values between the LN and non-LN SLE groups (*p* > 0.05). Detailed sonographic data are presented in Table 2.

### 3.3. Laboratory Findings and Disease Activity

While the SLE patients were examined during their inactive period, it was found that those with LN in the active disease had significantly higher disease activity and worse renal function when contrasted with the non-LN SLE group. Significant findings in the LN group revealed:

- Longer disease duration (*p* = 0.035).
- Higher SLEDAI-2K scores (median: 15 vs. 9, *p* < 0.001).
- Higher serum creatinine levels (*p* = 0.001) and lower eGFR values (*p* < 0.001).
- Lower complement component 3 levels (*p* = 0.004).

Table 3 presents the laboratory findings and disease activity scores obtained from the patients’ medical records during their most recent active flare, specifically aiming to reflect the severity of their inflammatory history. While the current values were within normal limits (inactive disease), the historical active phase data revealed that no significant differences were found between the LN and non-LN SLE groups regarding anti-dsDNA titers (*p* = 0.516) or complement component 4 levels (*p* = 0.647). During their historical active flare, it is noteworthy that eight patients (18.6%) in the non-LN SLE group exhibited a discordant pattern, presenting with significant physical examination findings despite the absence of serological abnormalities (normal anti-dsDNA and complement levels).

Regarding treatment protocols and disease activity indicators, the use of mycophenolate mofetil and cyclophosphamide was significantly more frequent in the LN group (*p* = 0.001; *p* < 0.001 respectively).

Table 4 presents the comparison of current laboratory findings and disease activity results in patients with SLE. Statistically significant differences were observed in renal function tests between the groups with and without LN. The median creatinine level was significantly higher in the group with LN (0.76 mg/dL) compared to the group without LN (0.67 mg/dL) (*p* = 0.004). Similarly, eGFR values were significantly lower in the group with LN (93.06 mL/min/1.73 m^2^) compared to the group without LN (106.60 mL/min/1.73 m^2^) (*p* < 0.001). However, no statistically significant differences were observed between the groups regarding Anti-dsDNA, C3, and C4 levels, or SLEDAI-2K scores (*p* > 0.05). Although the groups were statistically similar, individual analysis revealed that four patients exhibited a ‘Serologically Active Clinically Quiescent’ (SACQ) pattern, characterized by isolated serological activity (elevated anti-dsDNA and/or hypocomplementemia) in the absence of clinical findings.

### 3.4. Correlation Analysis

To assess the impact of prior inflammation on current tissue stiffness, we analyzed the relationship between SWE values and retrospective data recorded during the most recent active flare. This analysis revealed that renal stiffness was significantly associated with historical renal function markers and disease activity:

- Creatinine (Active Phase): Positive correlation with both right (rho = 0.25, *p* = 0.005) and left (rho = 0.21, *p* = 0.005) kidney stiffness.
- eGFR (Active Phase): Negative correlation with both right (rho = −0.24, *p* = 0.007) and left (rho = −0.23, *p* = 0.040) kidney stiffness.
- SLEDAI-2K (Active Phase): A weak to moderate positive correlation was observed with right (rho = 0.24, *p* = 0.048) and left (rho = 0.23, *p* = 0.041) kidney stiffness.

No significant correlation was found between renal stiffness and historical anti-dsDNA levels (*p* > 0.05).

In contrast, as summarized in Table 5, Spearman correlation analysis revealed no statistically significant associations between renal SWE findings (stiffness or RI values) and current laboratory findings obtained during the inactive disease (including current renal function markers, complement levels, anti-dsDNA titers, or SLEDAI-2K scores) (all *p* > 0.05). This dissociation suggests that renal stiffness reflects chronic damage accrued from past activity rather than current inflammatory status.

## 4. Discussion

We aimed to investigate the diagnostic utility of SWE versus DUS parameters in detecting subclinical renal damage, specifically targeting SLE patients with LN in the inactive disease. The most striking finding of our study is that renal parenchymal stiffness (kPa) values in patients with LN were statistically significantly higher compared to both SLE patients without renal involvement and healthy controls. Critically, this elevation in stiffness was evident despite the entire LN cohort being in the inactive disease (clinical remission). Conversely, the DUS parameter (RI) did not show a significant difference between the groups. These results suggest that SWE is a more sensitive tool than RI for detecting early parenchymal changes, such as inflammation and fibrosis, that persist even during the quiescent disease state.

The pathological mechanism of LN involves immune complex deposition, complement activation and subsequent interstitial inflammation [2]. Over time, these processes lead to renal fibrosis, which increases tissue stiffness. SWE has been found to have significant diagnostic value in the evaluation of renal histopathology and is considered a reliable alternative for measuring tissue stiffness. An increase in tissue stiffness has been found to be associated with histological changes such as glomerular sclerosis, interstitial fibrosis, and tubular atrophy [17]. SWE can be useful not only in assessing kidney involvement but also in follow-ups to evaluate the progression of CKD in patients. A particular strength of this study lies in the distinct separation of the LN group from both healthy controls and non-LN SLE patients. This confirms that the observed increase in renal stiffness is not simply a manifestation of systemic inflammation but is indicative of intrinsic renal parenchymal pathology. Thus, our findings add valuable evidence to the limited literature on the clinical utility of SWE for differentiating LN from non-LN cases.

Our findings are consistent with studies in the literature investigating the use of SWE in other renal diseases. Studies on CKD and diabetic nephropathy have reported that SWE values correlate with the level of fibrosis and may offer earlier detection compared to standard tests [22,23,24,25].

SWE studies on different inflammatory diseases such as familial Mediterranean fever (FMF) also revealed promising results. SWE conducted on FMF patients has demonstrated significantly increased renal stiffness compared to healthy controls [26,27]. In particular, the positive correlation of renal stiffness with proteinuria and CRP levels parallels the positive correlation we observed with SLEDAI scores during the active disease [26]. Despite the distinct underlying pathology (LN/Fibrosis vs. FMF/Amyloidosis), our results are consistent with the literature on other autoinflammatory diseases regarding the detectability of renal damage through SWE. These findings bolster the hypothesis that SWE could act as a universal tool for assessing renal involvement in inflammatory diseases.

In our study, renal stiffness showed a significant positive correlation with serum creatinine and SLEDAI scores during the active disease, and a negative correlation with eGFR. This is consistent with the study by Chen et al. [23], which found that renal elasticity values in CKD patients correlated strongly with renal fibrosis scores and eGFR levels. Additionally, Leong et al. [17] emphasized that non-invasive imaging could mirror histological severity. Our data supports these current findings by demonstrating that during the most recent active phase, renal stiffness increases as renal function deteriorates and systemic disease activity rises (higher SLEDAI-2K).

Interestingly, while SWE successfully differentiated the LN group, RI measured by Doppler US failed to show significant differences between groups. Although RI is widely used to assess renal vascular resistance, its sensitivity in early-stage renal disease is debated. Jesrani et al. [28] and He et al. [29] have suggested that RI may not change significantly until advanced renal damage or significant vascular sclerosis occurs. Our results support the view that SWE is superior to DUS in the early detection of renal involvement, as it directly assesses tissue mechanical properties rather than hemodynamic changes, which may be compensated in the early stages of the disease.

A key contribution of this study is the shift in focus from acute inflammatory changes to permanent structural damage. In LN, the kidney undergoes a pathological transition from acute activity—characterized by inflammation and edema—to chronicity, marked by fibrosis and scarring. By correlating SWE values obtained during the inactive disease with SLEDAI-2K scores from the most recent active disease, we demonstrate that the severity of prior inflammation significantly dictates the degree of long-term tissue remodeling.

Building on this relationship between past inflammation and current remodeling, our findings suggest that while clinical remission involves the resolution of inflammatory edema and the normalization of urinary markers, a ‘stiffness legacy’ may persist in the renal parenchyma. Consequently, SWE may serve as a non-invasive surrogate for the Chronicity Index, capturing cumulative damage from prior high-activity flares that standard laboratory tests might overlook during periods of clinical quiescence. This highlights the potential of SWE to reflect the long-term structural impact of the disease beyond immediate clinical status.

While the differences in SWE between the LN and non-LN groups achieved statistical significance (*p* < 0.05), we acknowledge that the absolute differences in stiffness values are relatively narrow. This overlap suggests that SWE may be more valuable as a longitudinal monitoring tool for individual patients—tracking changes in stiffness over time—rather than as a standalone tool for the initial differentiation of LN from non-LN cases. The small absolute difference may also reflect the study population being in the inactive disease, where the absence of acute inflammatory edema narrows the stiffness gap between groups.

Our study has several limitations that should be acknowledged. First, the sample size was relatively small compared to multicenter studies conducted over longer periods. However, as a tertiary referral center receiving patients from multiple regions, and given the substantial number of patients recruited within a relatively short timeframe, we believe the results are representative and meaningful for the current patient population. Second, due to ethical constraints and the invasive nature of the procedure, renal biopsy could not be performed on healthy controls or non-LN SLE patients for histopathological correlation; therefore, comparisons relied on clinical and laboratory classifications.

Third, the cross-sectional design of the study limits the assessment of temporal changes. Longitudinal studies are needed to determine whether SWE can effectively track the response to immunosuppressive therapy over time. Nonetheless, this study highlights the clinical utility of the technique, providing a foundational basis for future longitudinal research. Fourth, SWE measurements are not entirely independent of operator experience or patient body habitus. In particular, increased skin-to-kidney distance in obese patients may compromise signal quality due to attenuation. Additionally, while the absence of significant differences in DUS parameters (RI) suggests limited sensitivity for detecting early-stage or mild involvement, the potential utility of RI in advanced or chronic disease stages should not be disregarded. Moreover, although the study groups were matched for age and comorbidities, the difference in disease duration between the groups is a limitation that should be considered when interpreting the results. Furthermore, although patients with hypertension and diabetes were only included if they were normotensive and euglycemic at the time of evaluation, this single assessment does not reflect long-term disease control. Cumulative damage from these comorbidities could act as confounding factors. Due to the limited sample size, multivariate analysis to adjust for these confounders could not be performed, which represents a limitation in isolating the independent effect of SWE.

Finally, the relatively small absolute differences in stiffness values between the groups could be viewed as a limitation for clinical differentiation. However, the high intra- and inter-observer reproducibility of SWE in renal applications, characterized by Intraclass Correlation Coefficients generally exceeding 0.80, ensures that these findings are technically robust [30]. This precision suggests that even small increments in stiffness may reflect meaningful underlying tissue remodeling rather than measurement error, supporting the validity of SWE in monitoring chronic progression in LN patients.

## 5. Conclusions

This study demonstrates that SWE is capable of detecting subclinical renal abnormalities—specifically signs of remodeling and fibrosis—in LN patients, even during the inactive disease. Unlike DUS (RI), SWE revealed elevated renal stiffness independent of acute inflammatory edema. These findings suggest that SWE holds potential as a non-invasive surrogate for assessing cumulative chronic damage that standard laboratory tests might overlook. However, while these cross-sectional results are promising, they should be interpreted with caution regarding longitudinal monitoring. Future studies involving histopathological correlation and a longitudinal design are required to definitively validate the utility of SWE in tracking disease progression over time. If confirmed, SWE could eventually complement routine follow-ups as a radiation-free tool, particularly for women of reproductive age, potentially reducing the need for invasive biopsies.

## Figures and Tables

**Table 1:** Demographic and Clinical Characteristics of Patient and Control Groups.

Variable	LN Group (n = 37)	Non-LN SLE Group (n = 43)	Control Group (n = 41)	*p*-Value
Age, years (median (IQR))	44 (15.5)	44 (22)	42 (17.5)	0.268 *
Gender				0.431 **
Male, n (%)	3	1	2	
Female, n (%)	34	42	39	
Duration of illness, months (median (IQR)) Presence of co-morbidities, n (%)	112.0 (126.0)	78.0 (110.0)	**-**	0.035 ***
Diabetes mellitus	1 (2.7)	1 (2.3)	-	1.000 **
Hypertension	6 (16.2)	7 (16.3)	-	1.000 **
Coronary Artery Disease	0 (0)	1 (2.3)	-	1.000 **
Hyperlipidemia	0 (0)	2 (4.7)	-	0.497 **
Obesity	2 (5.4)	2 (4.7)	-	1.000 **

LN: Lupus nephritis; SLE: Systemic Lupus Erythematosus; IQR: Interquartile Range; * Kruskal–Wallis test; ** Fisher’s Exact Test; *** Mann–Whitney-U test.

**Table 2:** SWE and resistive index values of SLE patients and control group.

Variable, Median (IQR)	LN Group (n = 37)	Non-LN SLE Group (n = 43)	Control Group (n = 41)	*p*-Value
Right kidney stiffness (kPa)	1.60 (0.40)	1.40 (0.29)	1.32 (0.18)	<0.001 *
Left kidney stiffness (kPa)	1.70 (0.38)	1.40 (0.22)	1.30 (0.12)	<0.001 *
Right kidney resistive index	0.62 (0.10)	0.62 (0.05)	0.60 (0.04)	0.083 *
Left kidney resistive index	0.60 (0.09)	0.62 (0.06)	0.60 (0.04)	0.328 *

LN: Lupus nephritis; SLE: Systemic Lupus Erythematosus; IQR: Interquartile Range; * Kruskal–Wallis test.

**Table 3:** Laboratory findings and disease activity scores of SLE patients recorded during their most recent active disease flare.

Variable, Median (IQR)	LN Group (n = 37)	Non-LN SLE Group (n = 43)	*p*-Value
Anti-dsDNA (IU/mL)	210.0 (366.0)	145.0 (360.0)	0.516 *
Creatinine (mg/dL)	1.13 (1.14)	0.74 (0.35)	0.001 *
eGFR (mL/min/1.73 m^2^)	55.09 (62.58)	98.00 (22.16)	<0.001 *
SLEDAI-2K	15.0 (11.5)	9.0 (5.0)	<0.001 *

LN: Lupus nephritis; SLE: Systemic Lupus Erythematosus; Anti-dsDNA: Anti-double-stranded DNA; SLEDAI-2K: Systemic lupus erythematosus disease activity index 2000; eGFR: estimated Glomerular filtration rate; IQR: Interquartile Range; * Mann–Whitney-U test.

**Table 4:** Comparison of current laboratory findings and disease activity scores in SLE patients with and without LN.

Variable, Median (IQR)	LN Group (n = 37)	Non-LN SLE Group (n = 43)	*p*-Value
Anti-dsDNA (IU/mL)	<10 (56.0)	12.8 (96.5)	0.460 *
Creatinine (mg/dL)	0.76 (0.42)	0.67 (0.11)	0.004 *
eGFR (mL/min/1.73 m^2^)	93.06 (43.15)	106.60 (22.66)	<0.001 *
C3 level (g/L)	0.97 (0.22)	0.98 (0.18)	0.602 *
C4 level (g/L)	0.20 (0.11)	0.19 (0.12)	0.481 *
SLEDAI-2K	0 (2)	0 (2)	0.928 *

LN: Lupus nephritis; SLE: Systemic Lupus Erythematosus; Anti-dsDNA: Anti-double-stranded DNA; SLEDAI-2K: Systemic lupus erythematosus disease activity index 2000; eGFR: estimated Glomerular filtration rate; IQR: Interquartile Range; * Mann–Whitney-U test.

**Table 5:** Correlation analysis of renal stiffness and resistive index with current laboratory findings and disease activity scores in patients with SLE.

r (*p*)	eGFR (IU/mL)	Creatinine (mg/dL)	C3 (g/L)	C4 (g/L)	Anti-dsDNA (IU/mL)	SLEDAI-2K
Right kidney stiffness (kPa)	−0.132 (0.245)	0.072 (0.526)	0.146 (0.196)	0.087 (0.441)	−0.050 (0.660)	−0.027 (0.812)
Left kidney stiffness (kPa)	−0.168 (0.137)	0.113 (0.317)	0.185 (0.100)	0.077 (0.495)	−0.091 (0.424)	−0.113 (0.316)
Right kidney resistive index	−0.146 (0.196)	0.096 (0.399)	0.030 (0.792)	0.219 (0.051)	−0.008 (0.944)	−0.002 (0.983)
Left kidney resistive index	−0.159 (0.159)	0.064 (0.574)	0.147 (0.194)	0.215 (0.055)	−0.015 (0.898)	0.020 (0.859)

SLE: Systemic Lupus Erythematosus; Anti-dsDNA: Anti-double-stranded DNA; SLEDAI-2K: Systemic lupus erythematosus disease activity index 2000; eGFR: estimated Glomerular filtration rate; r: Spearman correlation coefficient.

## Data Availability

Data supporting the findings of this study can be obtained, but there are restrictions on the availability of these data. These data were used under license for the current study and are therefore not publicly available. However, data can be obtained from the corresponding author upon reasonable request.

## Abbreviations

ANA	Antinuclear Antibody
Anti-dsDNA	Anti-double-stranded DNA
CKD	Chronic Kidney Disease
CRP	C-reactive protein
DUS	Doppler Ultrasonography
eGFR	estimated Glomerular Filtration Rate
ESRD	End-Stage Renal Disease
FMF	Familial Mediterranean Fever
IQR	Interquartile Range
kPa	Kilopascal
LN	Lupus Nephritis
RI	Resistive Index
ROI	Region of Interest
SLE	Systemic Lupus Erythematosus
SLEDAI-2K	Systemic Lupus Erythematosus Disease Activity Index 2000
SWE	Shear Wave Elastography
US	Ultrasonography
